# Microbial rhamnolipid production: a critical re-evaluation of published data and suggested future publication criteria

**DOI:** 10.1007/s00253-017-8262-0

**Published:** 2017-04-06

**Authors:** Victor U. Irorere, Lakshmi Tripathi, Roger Marchant, Stephen McClean, Ibrahim M. Banat

**Affiliations:** School of Biomedical Sciences, Faculty of Life and Health Sciences, Ulster University, Coleraine, Co. Londonderry BT52 1SA UK

**Keywords:** Biosurfactants, Rhamnolipid, Rhamnolipid genes, Microbial identification, Biosurfactant quantification

## Abstract

High production cost and potential pathogenicity of *Pseudomonas aeruginosa*, commonly used for rhamnolipid synthesis, have led to extensive research for safer producing strains and cost-effective production methods. This has resulted in numerous research publications claiming new non-pathogenic producing strains and novel production techniques many of which are unfortunately without proper characterisation of product and/or producing strain/s. Genes responsible for rhamnolipid production have only been confirmed in *P. aeruginosa*, *Burkholderia thailandensis* and *Burkholderia pseudomallei*. Comparing yields in different publications is also generally unreliable especially when different methodologies were used for rhamnolipid quantification. After reviewing the literature in this area, we strongly feel that numerous research outputs have insufficient evidence to support claims of rhamnolipid-producing strains and/or yields. We therefore recommend that standards should be set for reporting new rhamnolipid-producing strains and production yields. These should include (1) molecular and bioinformatic tools to fully characterise new microbial isolates and confirm the presence of the rhamnolipid *rhl* genes for all bacterial strains, (2) using gravimetric methods to quantify crude yields and (3) use of a calibrated method (high-performance liquid chromatography or ultra-performance liquid chromatography) for absolute quantitative yield determination.

## Introduction

The use of synthetic or petrochemical-based surfactants has received considerable criticism in recent times due to their low biodegradability and environmental toxicity (Hrenovic and Ivankovic 2007; Reis et al. 2013; Scott and Jones 2000). Additionally, the use of rapidly depleting petrochemical resources in the production of synthetic surfactant is a source of some concern. These concerns have driven the search for sustainable substitutes of low toxicity obtained from renewable sources to replace synthetic surfactants. Biosurfactants, a group of naturally produced surface active compounds, are presently considered as potential substitutes for synthetic surfactants due to their biodegradability, low toxicity and ability to be produced from renewable sources of raw materials (De Almeida et al. 2016; Uzoigwe et al. 2015).

Like their synthetic counterparts, biosurfactants are surface active molecules possessing both hydrophobic and hydrophilic moieties (Banat et al. 2010; Mulligan et al. 2001; Smyth et al. 2014). They are synthesised by a wide range of microorganisms (such as bacteria, yeast and fungi) as secondary metabolites with some biosurfactants playing essential roles (such as facilitating nutrient transportation and microbe host interaction) needed for the survival of these microorganisms (Rodrigues et al. 2006; Vasileva-Tonkova et al. 2006). Different types of biosurfactants have been identified, and these have been classified into two major categories: low molecular and high molecular weight biosurfactants. The glycolipids form a class of low molecular weight biosurfactants containing some of the most studied types of biosurfactants that have shown excellent promise for commercial production and utilisation (Gautam and Tyagi 2006; Kitamoto et al. 2002; Marchant and Banat 2012a, 2012b). They are typically made up of carbohydrate molecules linked to long-chain aliphatic or hydroxyaliphatic fatty acids (Gautam and Tyagi 2006; Marchant and Banat 2012a). Within this group, rhamnolipids are some of the most promising.

As the name implies, rhamnolipids comprise rhamnose unit/units linked to 3-hydroxyl fatty acid unit/units via β-glycosidic bond. The rhamnose units are linked to each other by O-glycosidic bonds, while the 3-hydroxyl fatty acids are linked to each other by an ester bond. Rhamnolipids occur as mono-rhamnolipid and di-rhamnolipid based on the number of rhamnose units within the molecule and are further differentiated into various congeners depending on the composition of the fatty acid units (Abdel-Mawgoud et al. 2011; Abdel-Mawgoud et al. 2010; Irfan-Maqsood and Seddiq-Shams 2014).

Presently, rhamnolipids are produced mainly by *Pseudomonas aeruginosa* by various companies for commercial purposes (Sekhon Randhawa and Rahman 2014). They also have potential applications in a range of industries including medical, environmental, agricultural, cosmetics and pharmaceutical industries (Table 1). However, the high cost of production compared to synthetic surfactants has to a large extent hindered widespread application (De et al. 2015). As a result, present research has focused largely on identifying possible ways of reducing the cost of rhamnolipid production including the use of cheap substrates and identifying strains with high production capacity. Furthermore, the main production organism for rhamnolipid, *P. aeruginosa*, is classified as an opportunistic or ‘group II’ pathogen. This has raised various concerns including the risk of opportunistic infection by *P. aeruginosa* during large-scale industrial production (Neto et al. 2009) and the safety of the synthesised material, especially in applications involving human contact such as biomedical, cosmetics and pharmaceutical applications (Uzoigwe et al. 2015). As a result, researchers have also focused their attention on the search for suitable alternative non-pathogenic or ‘safe’ microorganisms capable of producing rhamnolipids.

**Table 1:** Potential applications of rhamnolipid in different industries

Industry	Application	Reference
Biomedical	Antimicrobial and/or antiviral agents	Cortés-Sánchez et al. (2013), Remichkova et al. (2008)
	Treatment of autoimmune disease	Piljac and Piljac (1995)
	Wound healing, treatment of gum disease and periodontal regeneration	Stipcevic et al. (2006)
	Treatment of ulcer	Piljac et al. (2008)
Environmental	Biodegradation of organic compounds	Maier and Soberón-Chávez (2000)
	Adsorption and composting of heavy metals	Fu et al. (2015)
	Environmental disinfection and cleaning	DeSanto (2012)
Cosmetics and pharmaceutical industries	Treatment of radiation burns	DeSanto (2011)
	Treatment of wrinkles and signs of ageing	Piljac and Piljac (2007)
	Used in antidandruff products, nail care products and toothpaste	Lourith and Kanlayavattanakul (2009)
Other industries	It is used in the food industry as a multipurpose ingredient as well as serving as a source of L-rhamnose and conditioning of food surfaces	Rikalović et al. (2015)
	In the agricultural industries, it is used for remediating agricultural soils, improving microbial plant interaction, improving plant nutrient absorption, as biopesticides and as antimicrobial agents for farm animals	Rikalović et al. (2015), Sekhon Randhawa and Rahman (2014)
	It is also used in nanotechnology for the formulation of microemulsion and synthesis of nanoparticles as well as in the formulations of drug delivery systems	Rikalović et al. (2015)

The need for less expensive and safer routes for rhamnolipid production has resulted in the publication of increasing numbers of research papers on rhamnolipid production. However, many of the published works are generally unreliable, largely due to insufficient data to support the identity of the producing organism, incomplete characterisation of the product as well as poor methodology. This review is therefore aimed at evaluating the published literature on rhamnolipid production and recommending minimum guidelines for acceptance of future publications on rhamnolipid production.

## Microbial rhamnolipid producers

*P. aeruginosa* (previously known as *Pseudomonas pyocyanea*) was the first reported rhamnolipid producer in the mid-twentieth century (Bergström et al. 1946). Throughout most of the twentieth century, reports on rhamnolipid production were generally from strains of *Pseudomonas* spp., essentially *P. aeruginosa* (Abdel-Mawgoud et al. 2010). Most of the research around this time was aimed at understanding the chemical structure of rhamnolipids as well as elucidating their function and mechanism of biosynthesis and regulation (Burger et al. 1963; Burger et al. 1966; Edwards and Hayashi 1965; Guerra-Santos et al. 1986; Hauser and Karnovsky 1957; Jarvis and Johnson 1949; Koch et al. 1991; Ochsner et al. 1994). However, as the need for increased production and safer microbial producers became a topic of major discussion, researchers started reporting various rhamnolipid-producing strains other than *P. aeruginosa* as well as strains in genera other than *Pseudomonas*.

A standard literature search on any of the databases will produce a list of publications with claims of biosurfactant or rhamnolipid production by various organisms, the majority of which were isolated from natural or artificial soil/water samples or industrial facilities (Kaya et al. 2014; Liu et al. 2013; Lotfabad et al. 2009; Pimienta et al. 1997; Roy et al. 2015; Saravanan and Vijayakumar 2012; Sharma et al. 2014; Wasoh 2013). However, one major problem identified in some of these cases was the lack of suitable molecular characterisation techniques for the identification of these microorganisms (Marchant et al. 2014).

Some reports based their identification mainly on physical methods of microbial characterisation such as gram staining, morphological appearances, Biolog GEN III, the analytical profile index (API) test as well as other biochemical test for microbial classification (Table 2). While these tests might be useful for quick and routine microbial characterisation, they are not sufficient for conclusive microbial identification. This is largely because the expression of certain phenotypic characteristics can vary with environmental conditions (Janda and Abbott 2002; Rossello-Mora and Amann 2001), thus leading to false positive/negative results of phenotypic traits and consequently wrong assignment of the isolated strains.

**Table 2:** Microorganisms with claims of rhamnolipid production, including method of microbial identification and rhamnolipid characterisation

Reference	Organism	Organism identification method(s)	Biosurfactant type	Characterisation method	Biosurfactant/rhamnolipid yield (mg/L)	Yield determination method(s)	*rhl* genes identified
El-Amine et al. (2012)	*P. fluorescens* P.V:10	Phenotypic identification, API 20E strip, using selective media and fluorescence properties	Rhamnolipid	NA	105.756 ± 2.076	Rhamnose measurement by UV spectroscopy	NA
	Additional information/remarks: No rhamnolipid extraction recorded with supernatant used to test emulsification activity, oil displacement and drop collapse. Insufficient evidence to support rhamnolipid production, yield and isolate characterisation.						
Nalini and Parthasarathi (2014)	*Serratia rubideae*	16S rRNA sequencing	Rhamnolipid	GC-MS, FTIR, thin-layer chromatography	Not indicated	Emulsification index	*rhl*A only
	Additional information/remarks: Rhamnolipid was extracted, but its weight was not recorded. Rha-Rha-C_16_-C_10_ and Rha-Rha-C_14_-C_10_ were reported as the major rhamnolipid congeners. Requires further molecular evidence to identify *rhl* genes in isolate.						
Nordin et al. (2013)	*Pseudomonas* sp.	Phenotypic and morphological techniques, Biolog GEN III	No specific Bs identified	NA	432	Gravimetric	NA
	Additional information/remarks: Oil spreading, drop collapse test and emulsification index were used as evidence of biosurfactant production. Requires further evidence to support claim including the presence of *rhl* genes and identification of at least one rhamnolipid congener. Also requires further molecular evidence to support microbial characterisation such as 16S rDNA sequencing.						
Gunther et al. (2005)	*P. chlororaphis*	Referenced as identified previously by Stanier et al. (1966). Identification by morphological appearance and biochemical reaction.	Rhamnolipid	TLC and HPLC/MS	>355	Gravimetric	NA
	Additional information/remarks: Only mono-rhamnolipid congeners identified: Rha-C_10_-C_10_, Rha-C_12_-C_10_, Rha-C_12:1_-C_10_, Rha-C_12_-C_12_, Rha-C_12:1_-C_12_, Rha-C_14_-C_10_, Rha-C_14:1_-C_10_ and Rha-C_10_-C_8_. Requires 16S rDNA characterisation of producing organism, also requires molecular evidence to support claims of rhamnolipid production.						
Vasileva-Tonkova et al. (2006)	*P. fluorescens* HW6	Morphological and biochemical identification following the Bergey’s Manual of Determinative Bacteriology.	Glycolipid ‘assumed to be rhamnolipid’	Haemolysis, growth inhibition of *Bacillus subtilis* and TLC	1400 ± 220	Orcinol assay on extracted product	NA
	Additional information/remarks: It decreased the surface tension of water to 35 mN/m with critical micelle concentration of 20 mg/L. Requires 16S rDNA sequencing for isolate identification, need to show at least one rhamnolipid congener and need to show molecular evidence (presence of *rhl* genes) to support rhamnolipid production claim.						
Tuleva Borjana et al. (2002) and (Martinez-Toledo et al. 2006)	*P. putida*	Morphological and biochemical characterisation using the Bergey’s manual.	Rhamnolipid	Haemolysis, growth inhibition of *Bacillus subtilis* and TLC. IR spectra of the supernatant were also used as evidence of rhamnolipid production	1200	Orcinol assay on extracted product	NA
	Additional information/remark: The surface tension of the media was reported to be reduced to 29 mN/m with emulsifying activity of kerosene reaching up to 69%. Requires 16S rDNA sequencing for isolate identification, need to show at least one rhamnolipid congener and need to show molecular evidence (presence of *rhl* genes) to support rhamnolipid production claim.						
Priji et al. (2017)	*Pseudomonas* sp. BUP6	16S rRNA sequencing	Rhamnolipid type assumed to be rhamnolipid	FTIR, TLC, orcinol	2070	Gravimetric	NA
	Additional information/remark: Minimum surface tension observed 35–40 mN/m. No determination of congener composition or definitive identification of biosurfactant. Need to show at least one rhamnolipid congener and need to show molecular evidence (presence of *rhl* genes) to support rhamnolipid production claim.						
Rooney et al. (2009)	*Enterobacter hormaechei* NRRL B-59185; *E*. *asburiae* NRRL B-59189; *Acinetobacter calcoaceticus* NRRL B-59190; *A. calcoaceticus* NRRL B-59191; *Pantoea stewartii* NRRL B-59187	16S rRNA sequencing	Rhamnolipid	Matrix-assisted laser desorption/ionisation time-of-flight MS (MALDI-TOF/MS)	1900 to 2400	Gravimetric	NA
	Additional information/remark: The strains were isolated alongside strains of *P. aeruginosa*. Yield obtained and congener composition were comparable to that obtained using the isolated *P. aeruginosa* strains with the exception of *A. calcoaceticus* NRRL B-59191 which had only Rh_2_-C_10_-C_10_ and Rha-C_10_-C_10_ congeners. Requires identification of *rhl* genes. This may further help explain the reason for similar congeners observed as those observed in *P. aeruginosa* and support the possibility of a lateral transfer of genes explained in this review.						
Dubeau et al. (2009)	*B. thailandensis* E264	16s rDNA, referencing (Brett et al. 1998)	Rhamnolipid	HPLC/MS/MS	∼260	LC/MS	Yes
	Additional information/remark: The *rhlA*, *rhlB* and *rhlC* genes were reported to be contained within a single operon compared to *P. aeruginosa* PAO1 in which *rhl*C is located in a different operon from *rhl*AB. Reported CMC was >200 mg/L. The authors suggested that the presence of large chain congeners (Rha_2_ C_14_-C_14_ and C_14_-C_16_) may be the reason for this attribute. Yield >2700 mg/L have recently been reported using gravimetric quantification (Funston et al. 2016).						
Christova et al. (2004)	*Renibacterium salmoninarum* 27BN	Physiological and biochemical tests following the Bergey’s manual	Rhamnolipid	TLC and infrared spectrometer	Up to 920	Orcinol assay	NA
	Additional information/remarks: The IR spectra were compared to that obtained from commercially available rhamnolipids for conclusive evidence on rhamnolipid production. Need to show at least one rhamnolipid congener and need to show molecular evidence (presence of *rhl* genes) to support rhamnolipid production claim. Requires 16S rDNA for proper characterisation of microbial isolate.						
Tavares et al. (2013)	*B. kururiensis* KP23^T^	Previously identified using various physiological, biochemical and molecular techniques (Zhang et al. 2000)	Rhamnolipid	ESI-LTQ Orbitrap Hybrid MS	780	Orcinol assay with a conversion factor of 2.00	NA
	Additional information/remarks: Rha_2_-C_10_-C_10_ was identified as the prevalent congener with no species of C_14_-C_14_ reported. Also mono-fatty acid congeners such as Rha_2_-C_8_, C_10_ and C_12_ were also reported. Need molecular evidence to show the presence of *rhl* genes to further support rhamnolipid production claim.						
Dubeau et al. (2009), Häußler et al. (1998)	*B. pseudomallei*	Obtained from national collection	Rhamnolipid	HPLC/MS/MS, ESI-MS and NMR spectroscopy	Not specified	NA	Yes: gene structure and arrangement similar to that observed in *B. thailandensis* E264
	Additional information/remarks: Rha_2_-C_14_-C_14_ was considered to be the most prevalent rhamnolipid congener						

*NA* not available, *TLC* thin-layer chromatography, *ESI-MS* electrospray ionisation mass spectrometry, *FTIR* Fourier transform infrared spectrometry, *NMR* nuclear magnetic resonance, *HPLC/M*S high-performance liquid chromatography mass spectrometry

Furthermore, the use of commercially available kits to characterise microbial isolates is generally unreliable as some of these kits and their data may be outdated following identification of new species and reassignment of old ones. A clear example according to Janda and Abbott can be found in the API 20E strip, where the tests on the strip in 1975 were unchanged in 2001 (Janda and Abbott 2002).

In one case, the API 20E test and fluorescence properties were used to classify organisms identified as rhamnolipid producers as *P. aeruginosa* P.B:2 and *Pseudomonas fluorescens* P.V:10 (El-Amine et al. 2012). A recent report has however shown that isolates identified as *P. fluorescens* using the API 20E tests were actually *Pseudomonas synxantha* and *Pseudomonas brassicacearum* using 16S ribosomal DNA (rDNA) sequencing; these are species which are not listed on the API 20E database (Wellinghausen et al. 2005). It is certain that a comprehensive look at published reports will identify cases where isolates have been wrongly characterised; this is however beyond the scope of this review.

Sequencing 16S rDNA has greatly improved the identification and characterisation of rhamnolipid-producing isolates, but this does not come without its limitations. Previous research has suggested that 16S rDNA provides genus identification >90% in most cases and species identification at between 65 and 85% (Janda and Abbott 2007). However, in some cases where 16S identification has been used to assign species, significant phenotypic differences have been identified (Janda and Abbott 2007). This suggests that the sole use of molecular techniques to classify rhamnolipid microbial isolates is not sufficient and should be used in conjunction with suitable phenotypic and biochemical tests.

A further consideration is the quality of the 16S rDNA data used for the identification; many sequences lodged in the public databases are only partial sequences of varying lengths rather than the complete sequence generated from the cloned gene. Clearly, the shorter the partial sequence. the less reliable the identification will be.

Another problem identified in this area is that in some research where particular rhamnolipid-producing isolates have been classified as novel microbial strains, the 16S sequence of these strains is often not deposited in public databases (Zhang et al. 2000). This can lead to the assignment of multiple strain IDs to a single strain as isolates with <97% identity with strains available in public databases can be classified as new taxa (Janda and Abbott 2002).

Also, in some research where 16S rDNA sequences have been used to carry out microbial identification, results on multiple alignment and taxonomic classification are not presented either in the paper itself or in supplementary materials. In our laboratory, we have found cases of rhamnolipid-producing isolates reported as non-pathogenic strains of *Pseudomonas* to actually be *P. aeruginosa* strains. This is important because rhamnolipid production has in these cases been wrongly assigned to microbial species other than *P. aeruginosa*.

Furthermore, even when these isolates have been identified correctly, there is the problem of assigning rhamnolipid production to a particular strain without suitable molecular techniques to identify orthologs of the genes responsible for rhamnolipid production (*rhlA*, *rhlB* and *rhlC* orthologs) in these organisms (Marchant et al. 2014). Of all the organisms, other than *P. aeruginosa*, reported to produce rhamnolipid biosurfactant in the literature, only in *Burkholderia* spp., specifically *Burkholderia thailandensis* E264, *Burkholderia pseudomallei* and *Burkholderia glumae* BGR1, were the orthologs of *rhlA*, *rhlB* and *rhlC* identified (Costa et al. 2011; Dubeau et al. 2009) (Table 2). In these species, the three genes are grouped together in a single gene cluster which is in contrast to *P. aeruginosa* where *rhlC* is separate from *rhlA* and *rhlB* (Dubeau et al. 2009). Additionally, the gene cluster was found to be duplicated in the genomes of these organisms, except for *B. glumae*, with both clusters functioning in rhamnolipid production (Dubeau et al. 2009). This is interesting given that at least three species of *Burkholderia* have been reported to contain *rhl* genes with established rhamnolipid production ability. On the contrary, although the *Pseudomonads* have for long been considered the predominant rhamnolipid producers, only in *P. aeruginosa* have the genes responsible for rhamnolipid production been fully characterised.

Toribio et al. (2010) carried out a taxonomic analysis of *rhl* genes from four strains of *P. aeruginosa* (PAO1, PA14, LESB58, PA7), *B. thailandensis* and *B. pseudomallei*. They reported that the genes from these two genera belong to the same group in the phylogenetic tree, indicating that there is a low possibility of lateral gene transfer among the two genera. However, when they did a phylogenetic analysis to determine the presence of *rhlA*, *rhlB* and *rhlC* genes in other bacterial strains that have been reported to produce rhamnolipid, they did not find any sequence similarity in these organisms. Although, they used only those strains whose complete genome sequence is present in the public databases (Toribio et al. 2010). They further reported the presence of an *rhlA* ortholog in *P. fluorescens* SWB25, but no *rhlB* and *rhlC* orthologs were found, suggesting that *rhlA* might be involved in other pathways within the organism apart from rhamnolipid production (Toribio et al. 2010).

*Serratia rubidaea* SNAU02 was reported to be a rhamnolipid producer, and the report also claimed that the organism possesses rhamnolipid genes *rhlA* and *rhlB* (Nalini and Parthasarathi 2014). The authors reported that they sequenced the genes responsible for rhamnolipid production which they tagged *rhlAB* and deposited in the NCBI gene bank with accession number KF835609.1. They further reported that a blast search of the sequence showed similarity with the rhamnolipid genes of *P. aeruginosa* (Nalini and Parthasarathi 2014).

However, *rhlAB* is not a single gene but two separate genes present in a single operon in *P. aeruginosa*. A blast search of the gene sequence provided by the authors against the *P. aeruginosa* PAO1 genome indicates that this sequence is an ortholog of *rhlA* with 99% similarity; no similarity was found with *rhlB* or *rhlAB* operon. This shows that *S. rubidaea* probably only contains the *rhlA* ortholog with no evidence of *rhlB* and *rhlC*. Thus, there is not enough genetic evidence to support rhamnolipid production by *S. rubideae*.

The lack of orthologs of *rhl* genes in the genome sequence of these non-pseudomonas isolates available in public databases suggests that if rhamnolipid production is found in new isolates of these strains, it would have been acquired in the environment possibly through lateral transfer of genes (Toribio et al. 2010). This is most likely the case especially as these organisms are often isolated with rhamnolipid-producing organisms, particularly *P. aeruginosa* rhamnolipid-producing strains. It is therefore important that the *rhl* genes from reported rhamnolipid producers be sequenced and analysed to determine their origin. This is important for two main reasons: (i) It confirms the basis upon which rhamnolipid production is assigned and (ii) it indicates if rhamnolipid production has been acquired from the environment through gene transfer.

The second benefit is very crucial, as other researchers might want to obtain these strains from public culture collections and analyse them for rhamnolipid production. In some cases, where this has been done in our lab, we have found that these strains were not able to produce rhamnolipids. This suggested that either these strains have been wrongly identified or that isolate has acquired rhamnolipid production from the environment or the organism does not in fact produce rhamnolipids at all. Furthermore, isolated strains should be made available in public culture collections, so researchers can access these strains for further experiment. Although, it is understandable that this might not always be possible due to issues of intellectual property rights, industrial interest or patent filing.

## Rhamnolipid yield measurement and congener composition

Claims of high rhamnolipid yield are gradually accumulating in the literature in response to the need to reduce rhamnolipid production costs. While this is good for the future of possible rhamnolipid exploitation in commercial products, a major concern is the assignment of yield values to microbial isolates or fermentation processes compared to what has previously being published, without taking into consideration the differences in the methods used in analysing overall rhamnolipid yield or quantifying individual rhamnolipid congener composition.

Various approaches are presently used to improve the production of rhamnolipid, including identification of high rhamnolipid-producing isolates, varying carbon and nutrient sources, varying growth and fermentation conditions, optimising recovery techniques and genetic engineering of the producing microorganisms (Camilios Neto et al. 2008; Chen et al. 2007a; Giani et al. 1996; Mukherjee et al. 2006; Rooney et al. 2009; Soares dos Santos et al. 2016). While these techniques have recorded varying rhamnolipid yields with values >100 g/L (Giani et al. 1996), a look at the methods used in quantifying the recorded yield shows that each experiment has applied different methods to quantify rhamnolipid yield and composition (Table 2). This makes it difficult to compare these methods when looking for suitable techniques to improve rhamnolipid production or to improve a particular congener composition.

A review of the different techniques presently used in quantifying general yield of rhamnolipid and congener composition is beyond the scope of this study and can be assessed in the following references (Heyd et al. 2008; Smyth et al. 2014).

### Total rhamnolipid yield measurement

Rhamnolipid yields have been determined using a range of different methods; these include the use of simple gravimetric methods, colorimetric methods, infrared methods and high-performance liquid chromatography (HPLC) (Table 2). The gravimetric method is a direct and easy method to measure the weight of extracted rhamnolipid. It simply involves isolating and separating rhamnolipid and then measuring its weight.

This approach suffers from the major disadvantage that the isolated rhamnolipids are often not pure due to residual fatty acids or unspent carbon sources from the fermentation feedstock which may be extracted with the product (Abdel-Mawgoud et al. 2011; Marchant et al. 2014). This method also gives no indication of the purity of the ‘crude’ product being quantified. As stated by Marchant and Banat (2014), while solvent extraction can be used to remove most impurities, some fatty acids may not be removed and these impurities may also not be easily detected by spectrometric techniques.

Colorimetric methods have also been quite commonly used in rhamnolipid quantification. Several methods exist including orcinol assay, anthrone assay and the 6-deoxyhexose method (Smyth et al. 2014; Zhang and Miller 1992). Most colorimetric methods measure the amount of pentose sugar using a spectrophotometric assay coupled with a standard curve to quantify the amount of rhamnose in the sample which is then used to infer the quantity of the rhamnolipid by applying a multiplication factor.

These methods can also be used to measure the amount of rhamnolipid present within a fermentation broth without actually extracting the rhamnolipid, thus providing a means of monitoring yields during the fermentation processes. However, major limitations with these methods are the fact that the sugar determination is not specific for rhamnose and other sugars present will inflate the apparent value; rhamnose is present in compounds other than rhamnolipid in the cells, and finally, even with a correct determination of the quantity of rhamnose, the final estimate of rhamnolipid is made with a multiplication factor based on an estimate of the proportion of mono-rhamnolipid to di-rhamnolipid. From our experience of the orcinol assay, rhamnolipid yield values obtained from this method are always severely inflated compared to more robust quantification methods. This can result in huge discrepancies when comparing exact yields from fermentations involving different methods and microorganisms.

For example, when comparing *P. aeruginosa* strains known to predominantly produce Rha_2_-C_10_-C_10_, with *B. thailandensis* known to predominantly produce Rha_2_-C_14_-C_14_, only the rhamnose sugar will be measured. In this case, it would be inferred that the yield from both strains would be the same for each unit of rhamnose measured. However, this would be an incorrect value due to the additional eight-carbon units in each *B. thailandensis* rhamnolipid molecule.

Problems also occur when trying to compare yields from different experiments in which a colorimetric method was used in measuring rhamnolipid yield in one, while in others, different methods were used. For example, in their review to compare the yield of biosurfactants, including rhamnolipid, produced by different microbial strains using inexpensive carbon sources, Mukherjee et al. reported that *Pseudomonas* species DSM 2874 had the highest yield of 45 g/L while the other strains had yields <15 g/L (Mukherjee et al. 2006). However, close examination of the data gives a different interpretation. Trummler et al. (2003) who recorded the highest rhamnolipid yield with *Pseudomonas* species DSM 2874 had used a gravimetric method to estimate total rhamnolipid yield, with all the problems of uncertainty concerning the level of purity of the sample, while the other reports cited used different colorimetric methods to estimate yields. In one case, the authors of the report multiplied the rhamnose concentration by 3 in order to give an estimated total rhamnolipid yield (Nitschke et al. 2005).

In this latter case, their estimated rhamnolipid yield will be three times higher than other reported yields that have used similar colorimetric methods due to the use of the additional mathematical factor in the yield estimation. In the final analysis, a truly rigorous quantitative analysis of rhamnolipid yield can only be obtained using the type of protocol described by Rudden et al. (2015) where separation of a purified extract of rhamnolipids was carried out using ultra-performance liquid chromatography (UPLC) followed by tandem mass spectrometry to identify the individual congeners. A critical aspect of the method was the initial preparation of pure individual congener samples using flash chromatography which could then be used as quantified calibration samples. Using this method gave yield values that were considerably lower than other less accurate methods.

### Product identification

The reduction of surface and interfacial tensions, emulsification properties, haemolytic activities and binding to or reactions with dyes or cationic surfactants such as cetyltrimethylammonium bromide (CTAB) are all methods employed in the screening of microbial isolates for rhamnolipid production (Heyd et al. 2008; Marchant et al. 2014; Walter et al. 2010). A major advantage of these methods is that they are quick and cheap to perform, requiring little technical expertise. Additionally, the haemolytic test and CTAB test can be carried out before setting up any fermentation run and thus can help screen out a range of isolates, saving time and additional cost.

These advantages have made these techniques very useful during the early stages of rhamnolipid research. However, a major drawback of these methods is that they are non-specific and microorganisms manufacture a wide range of metabolites which have surface active properties with very similar reactions in these tests as rhamnolipid (Heyd et al. 2008), thus giving false positive results in the search for new rhamnolipid-producing isolates.

For example, haemolytic activities can be influenced by other lytic enzymes produced by microbial isolates (Siegmund and Wagner 1991). Although CTAB was developed to curb this major disadvantage of the haemolytic test, CTAB is a harmful substance and can also limit the growth of some microorganisms (Walter et al. 2010). It is also not specific for rhamnolipid as it can be used in detecting other glycolipid and anionic surfactants (Walter et al. 2010).

Despite their drawbacks, these methods are still very useful in screening for potential biosurfactant and/or rhamnolipid producers and fulfil the criteria set out by Chen et al. (2007b) for quick screening of biosurfactant producers, which includes the ability to screen many candidates quickly, the ability to assess quantitatively the effectiveness of the surfactants and the ability to identify potential organisms. However, they are not sufficient to provide conclusive evidence that a microbial isolate does produce rhamnolipid.

To address this issue, Walter et al. (2010) recommended a combination of different methods such as the drop assay, surface and interfacial tension, haemolysis, CTAB assay, oil spreading and microplate assay for a successful screening to overcome the advantages and disadvantages of each individual method. While this recommendation helps narrow down potential rhamnolipid producers from a wide range of isolates, it should not be used as conclusive evidence, that a particular isolate does produce biosurfactants/rhamnolipid. It is therefore important that necessary fermentation, isolation and purification steps be carried out followed by other analytical techniques (such as LC-MS or LC-MS/MS) to identify the particular type of biosurfactants/rhamnolipid produced. Therefore, for a particular isolate to be reported as a rhamnolipid producer, at least one rhamnolipid congener must have been identified (in addition to the identification of the *rhl* genes), using any of the techniques discussed below.

### Identifying rhamnolipid composition

The development of high-throughput techniques has enhanced our ability to analyse rhamnolipids. This has also led to an increase in the amount of rhamnolipid congeners known from just a few in the early 50s to over 60 (Abdel-Mawgoud et al. 2010). Techniques such as nuclear magnetic resonance (NMR), attenuated total reflectance-Fourier transform infrared spectroscopy (ATR-FTIR), liquid chromatography mass spectrometry (LC/MS) and LC/MS/MS help to precisely identify rhamnolipid and also partially quantify the various congeners present in a particular sample. A detailed description of these various techniques has previously being carried out (Leitermann et al. 2008; Smyth et al. 2014).

It is therefore important when reporting a particular isolate as a rhamnolipid producer that suitable techniques such as those mentioned above should be used to provide sufficient evidence that these isolates do produce rhamnolipid. As seen in Table 2, some reports that have claimed the production of rhamnolipid from environmental isolates have come to their conclusion using crude techniques such as haemolytic activities, CTAB assay, surface and interfacial properties and drop collapse assay, without any detailed chemical analysis of the extracts. These conclusions are not useful as results obtained from such crude analysis can only be indicative of some microbial surface active agent and not specifically rhamnolipid.

Therefore, biochemical characterisation of extracted microbial exudates presumed to be rhamnolipid biosurfactant should be carried out before it can be concluded that a particular isolate produces rhamnolipid or biosurfactant generally. Furthermore, the type of congener produced should also be reported together with their relative compositions. One advantage of knowing the congener composition is that it will help provide evidence to suggest if the particular isolate has acquired the rhamnolipid genes by lateral gene transfer. If lateral gene transfer is involved in the acquisition of rhamnolipid production ability by an isolate, the isolate will hypothetically produce rhamnolipid with congeners similar to the congeners produced by the original rhamnolipid producer from which the gene was acquired.

## Recommendation and conclusion

The global market for rhamnolipid production holds great promise (Sekhon Randhawa and Rahman 2014). This dictates the need for increased industrial production and safety of the production processes used particularly for the need for safer microbial strains. However, it is essential that guidelines be implemented for publications of research results particularly those with claims of isolating rhamnolipid producers with high production capacity and non-pathogenicity. Without a satisfactory level of scientific rigour in this area, the literature will become overcrowded with reports that cannot be effectively used or relied on to advance the field. This review has considered three major areas including identification of the organism, identification of the *rhl* genes within the isolates and characterisation and quantification of the product. Based on the literature reviewed, we recommend that the following standards be adopted for the acceptance of publications with claims of rhamnolipid production from microbial isolate.

- Identification of isolated strains should be carried out using routine physiological and biochemical characterisation techniques followed by 16S rDNA sequencing and phylogenetic characterisation of the isolate. The 16S rDNA sequence should wherever possible be a complete and not a partial sequence. Additional protocols for microbial identification including protocols and primers for 16S rDNA sequencing as well as protocols for phylogenetic characterisation of microbial isolates can be found in the literature (Bond et al. 2000; Stefanis et al. 2013). However, in cases where the microorganism has previously been fully characterised using at least the methods highlighted above, reference can be made to the previous study and the source of the organism stated.
- After an isolate has been identified, bioinformatic analysis of the identified strain should be carried out using data available in public databases such as the NCBI or EMBL-EBI. In situations where the identified strain is considered to be a novel strain or where there is no complete annotation of the genome, PCR analysis should be carried out using primers of known *rhl* genes (such as *P. aeruginosa* PAO1 or *B. thailandensis* E264) to amplify potential *rhl* genes followed by sequencing of the amplified genes.
- After suitable identification of the isolate and establishment of the presence of *rhl* genes have been made, fermentation experiments followed by product extraction and characterisation should be carried out. We recommend that before a particular isolate is reported to be a rhamnolipid producer, at least one rhamnolipid congener must be identified using LC/MS (using either online or offline MS) or other suitable analytical techniques such as those listed above or in the following papers (Leitermann et al. 2008; Rudden et al. 2015; Smyth et al. 2014).
- Irrespective of the quantification method used, we recommend that extracted rhamnolipid should be measured gravimetrically to allow for general yield comparison. The gravimetric determination should also be made in conjunction with analytical methods to establish the purity of the sample being analysed. More robust standard for quantitative measurement of yield should be a chromatographic separation method followed by mass spectrometry identification of individual congeners and quantification against individual congener standards. The effect of carbon source used in rhamnolipid production should also be taken into consideration especially in reviews.
